# Army and Navy ECHO Pain Telementoring Improves Clinician Opioid Prescribing for Military Patients: an Observational Cohort Study

**DOI:** 10.1007/s11606-018-4710-5

**Published:** 2018-10-31

**Authors:** Joanna G. Katzman, Clifford R. Qualls, William A. Satterfield, Martin Kistin, Keith Hofmann, Nina Greenberg, Robin Swift, George D. Comerci, Rebecca Fowler, Sanjeev Arora

**Affiliations:** 1 ECHO Institute, University of New Mexico, Albuquerque, NM USA; 2 Department of Statistics, University of New Mexico School of Medicine, Albuquerque, NM USA; 3 Defense Health Agency, Falls Church, VA USA; 4 University of New Mexico, Albuquerque, NM USA; 5 Kennell and Associates, Falls Church, VA USA; 6 Department of Mathematics, University of New Mexico, Albuquerque, NM USA; 7 Department of Internal Medicine, University of New Mexico School of Medicine, Albuquerque, NM USA; 8 Department of Psychiatry and Behavioral Sciences, University of New Mexico School of Medicine, Albuquerque, NM USA

**Keywords:** clinician education, project ECHO, telementoring, opioids, opioid overdose deaths, benzodiazepines

## Abstract

**Background:**

Opioid overdose deaths occur in civilian and military populations and are the leading cause of accidental death in the USA.

**Objective:**

To determine whether ECHO Pain telementoring regarding best practices in pain management and safe opioid prescribing yielded significant declines in opioid prescribing.

**Design:**

A 4-year observational cohort study at military medical treatment facilities worldwide.

**Participants:**

Patients included 54.6% females and 46.4% males whose primary care clinicians (PCCs) opted to participate in ECHO Pain; the comparison group included 39.9% females and 60.1% males whose PCCs opted not to participate in ECHO Pain.

**Intervention:**

PCCs attended 2-h weekly Chronic Pain and Opioid Management TeleECHO Clinic (ECHO Pain), which included pain and addiction didactics, case-based learning, and evidence-based recommendations. ECHO Pain sessions were offered 46 weeks per year. Attendance ranged from 1 to 3 sessions (47.7%), 4–19 (32.1%, or > 20 (20.2%).

**Main Measures:**

This study assessed whether clinician participation in Army and Navy Chronic Pain and Opioid Management TeleECHO Clinic (ECHO Pain) resulted in decreased prescription rates of opioid analgesics and co-prescribing of opioids and benzodiazepines. Measures included opioid prescriptions, morphine milligram equivalents (MME), and days of opioid and benzodiazepine co-prescribing per patient per year.

**Key Results:**

PCCs participating in ECHO Pain had greater percent declines than the comparison group in (a) annual opioid prescriptions per patient (− 23% vs. − 9%, *P* < 0.001), (b) average MME prescribed per patient/year (−28% vs. −7%, *p* < .02), (c) days of co-prescribed opioid and benzodiazepine per opioid user per year (−53% vs. −1%, *p* < .001), and (d) the number of opioid users (−20.2% vs. −8%,*p* < .001). Propensity scoring transformation–adjusted results were consistent with the opioid prescribing and MME results.

**Conclusions:**

Patients treated by PCCs who opted to participate in ECHO Pain had greater declines in opioid-related prescriptions than patients whose PCCs opted not to participate.

**Electronic supplementary material:**

The online version of this article (10.1007/s11606-018-4710-5) contains supplementary material, which is available to authorized users.

## INTRODUCTION

An estimated 100 million Americans suffer from chronic pain.^1^ In the USA, prescriptions for opioid analgesics quadrupled between 1999 and 2012.^2^ Prescribing behaviors associated with increased overdose risk include co-prescribing benzodiazepines and opioids and exceeding a daily dose of 50 morphine milligram equivalents (MME).^3, 4^ Approximately 175 people die everyday from drug-related deaths.^5^ Drug overdose deaths surpass injury deaths caused by motor vehicle accidents and firearms.^6^ The public health epidemics of chronic pain and drug overdose affect both civilian and military populations.^7–10^

Chronic pain, opioid use disorder (OUD), and post-traumatic stress disorder (PTSD) frequently occur together. Preventing these conditions is a high priority for the Department of Defense (DoD).^10–15^ Pain is a leading reason patients seek medical care and primary care clinicians (PCCs) are often the first points of contact.^16, 17^ Pain management and safe opioid prescribing education for civilian pre-licensure students and PCCs is not universally required, but is required for MHS clinicians.^18–21^

Opioid misuse is a problem for both military and civilian populations.^4^ When opioids are prescribed to patients with PTSD and other mental health diagnoses, this may increase their risk for adverse events.^11, 22, 23^ In 2009, the DoD recognized that the military needed an optimal pain management plan and prepared a Pain Management Task Force (PMTF) Report recommending an evidence-based approach to manage chronic pain across the Military Health System (MHS).^24^ Among several training platforms available for pain education, the U.S. Army chose the University of New Mexico Health Sciences Center (UNMHSC) Project ECHO (Extension for Community Healthcare Outcomes) model of Chronic Pain training for its rapid diffusion of pain management strategies.^25^

This observational cohort study assessed whether PCC participation in telementoring through the Chronic Pain and Opioid Management TeleECHO Clinic (ECHO Pain) improved pain management and safe opioid prescribing skills.

## METHODS

### The ECHO Model

Project ECHO is a lifelong learning and guided practice model of medical education and mentoring that uses a hub-and-spoke design to create knowledge networks. Expert teams at the hub use multi-point videoconferencing to conduct virtual learning sessions. Spoke attendees include physicians, advanced practice clinicians, and care teams. TeleECHO sessions run for 2 h weekly (96 total hours annually). Each session consists of a short, evidence-based didactic followed by case discussions intended to reduce variations in care.

Benefits for participating in ECHO Pain include no-cost continuing medical education (CME) credits and increased diagnostic, treatment, and referral skills related to multi-modal pain management and OUD.^26^ Clinicians learn prescribing behaviors that reduce opioid overdose risks including judicious use of opioids, identification of patients at risk of OUD, and risks of co-prescribing.^27–29^ Participating PCCs learn to provide specialty care which would otherwise be difficult to obtain for patients in their own communities.^30, 31^

### Army and Navy ECHO Pain Intervention

**Fig. 1:**
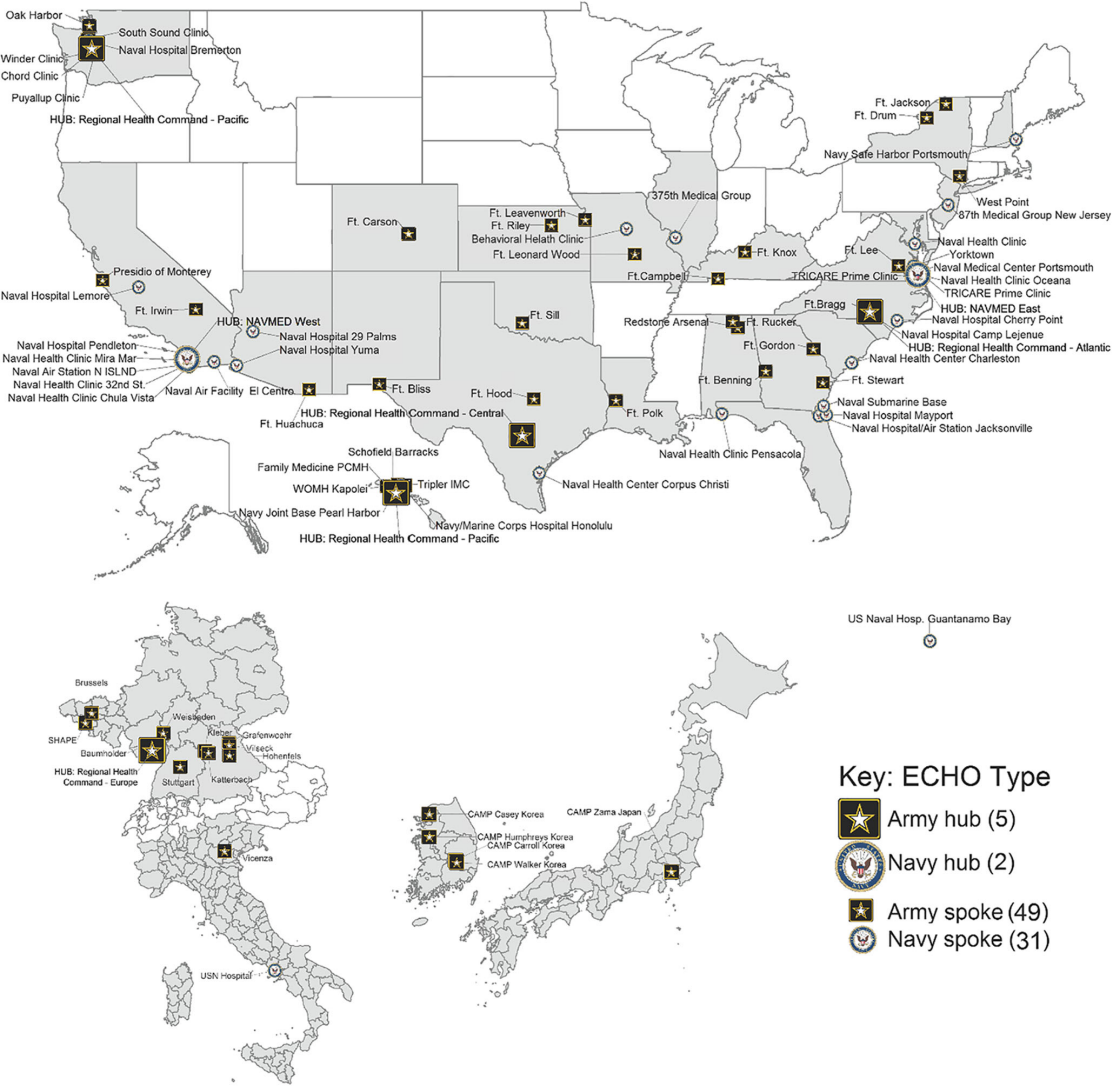
Geographic distribution of army and navy ECHO pain hubs and spokes.

Between 2012 and 2014, military medical treatment facilities (MTFs) were strategically chosen to serve as hub sites based on geographical location (time zone) and availability of specialty clinicians to serve as facilitators. Integrative and interdisciplinary pain teams were fully staffed at five U.S. Army and two U.S. Navy hub sites. The 47 remote Army and 33 remote Navy spoke locations were chosen based on PCC interest and volume of chronic pain patients**.** Project ECHO provided on-site and virtual trainings to both the hub and spoke clinicians regarding facilitation, case presentation skills, and the basics of pain management. See Fig. 1.

#### Study Design

**Fig. 2:**
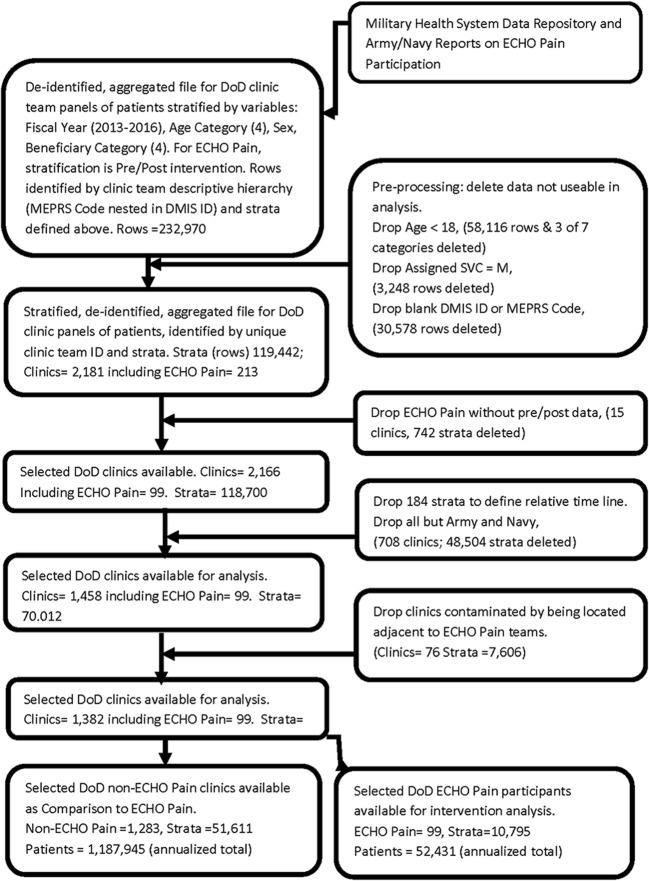
Flow diagram: data collection and analysis.

This is an observational cohort study comparing ECHO Pain participating PCCS with non-ECHO Pain participating PCCs. Clinics were separated into two primary groups: (1) 99 clinics whose PCCs voluntarily participated in ECHO Pain at least once per year and had data both before and after ECHO intervention and (2) 1283 comparison clinics that whose PCCs did not participate in ECHO Pain. Prescription counts for adult patients enrolled with Army and Navy PCC teams for fiscal years 2013 to 2016 (i.e., October through September) were supplied, de-identified, and aggregated from the Military Health System Data Repository (MDR). The MDR patient data was stratified by combinations of the covariables: fiscal year, age, sex, and beneficiary category and aggregated in these patient strata (combinations) for each clinic. Our study excluded patients under 18 years. Beneficiary categories included active duty military personnel, dependents of active duty personnel, members of the National Guard or Reserve, and military retirees. ECHO Pain data was additionally stratified for pre/post ECHO intervention. PCCs for ECHO Pain were either active duty or civilian clinicians working at Army or Navy MTFs (see Fig. 2).

### Primary and Secondary Outcomes

The primary outcome of this study was to assess whether voluntary PCC participation in ECHO Pain resulted in decreased prescriptions of opioids for enrolled patients. Secondary outcome measures included evaluation of MME dose and co-prescribing of opioids and benzodiazepines. Both primary and secondary outcome measures were developed prior to the launch of Army and Navy ECHO Pain. PCCs exposed to ECHO Pain (intervention group) were compared with PCCs who did not participate in ECHO Pain (comparison group).

### Outcome Variables

Prescriptions analyzed included opioids and their MME, benzodiazepines, and the overlap in the co-prescription of opioids and benzodiazepines. Opioids included codeine, dihydrocodeine, belladonna-opium, fentanyl, hydrocodone, hydromorphone, levorphanol, meperidine, morphine, methadone, oxycodone, tapentadol, and oxymorphone. Tramadol was also included as a partial opioid. Benzodiazepines included diazepam, lorazepam, clonazepam, alprazolam, temazepam, oxazepam, triazolam, midazolam, flurazepam, estazolam, quazepam, clorazepate dipotassium, and chlordiazepoxide/clinidium.

Covariables of age, sex, and beneficiary category were represented by the stratification used in the aggregation of data.

### Timelines

Onset of participation in ECHO Pain was staggered over 4 years to accommodate training in the ECHO model for the large number of Army and Navy hubs and spokes. Because ECHO Pain lacked a single start date, the authors matched the comparison groups with fiscal years to best analyze comparison and intervention groups. Demographics included assigned sex, age, service, and beneficiary category (see Aggregation in SOM**)**.

A common timeline was determined based on yearly midpoints which are labeled as −1.5 − 0.5, + 0.5, and + 1.5 relative to the starting time of ECHO Pain intervention (time = 0). For the comparison group, fiscal year midpoints were used and the first 2 years were labeled as − 1.5 and − 0.5 (see Definition of Time Line in Supplemental Online Material (SOM)).

### Analyses

Statistical significance was determined for rate changes over time, both within and between groups, evaluated as slopes using Repeated Measures (RM) Analysis of Covariance (ANCOVA) weighted by the numbers of patients. Log-transformed data were used so that slopes represent relative percent change. These percent changes are comparable.

Alternative analysis of primary outcomes used a propensity scoring transformation with ECHO Pain as the target distribution to reduce selection bias in the baseline values. Alternative analysis adjusted for sex, age, and beneficiary category as covariables (see Propensity Scoring sections in SOM).

Outcome variables were analyzed as a time series of clinic averages per patient. Opioid prescriptions were also analyzed for percentages of opioid users, and opioid prescriptions per opioid user, as these explanatory analyses are based on the decomposition of rates given by the formula: opioid prescriptions per enrollee = (opioid scripts/opioid users) × (opioid users/patients). MME rates and co-prescribed days of opioids and benzodiazepines were analyzed similarly.

### Institutional Review Board Approval

This study was approved by the UNMHSC Human Research Protections Office (study ID #16-388) and the Defense Health Agency (DHA) Institutional Review Board (CDO-16-2036 IRB #879675). A data sharing agreement (FP1032 DHA 17-1670) was signed by the DHA and UNMHSC to allow for data to be shared between the two institutions.

## RESULTS

### Demographics and Baseline Rates

The comparison group had 1283 clinics and ECHO Pain had 99 clinics. Baseline demographics for ECHO Pain and comparison clinics for sex, age, service, and beneficiary status as well as PCC participation level are given in Table 1.

**Table 1:** Baseline Demographics of Adult Study Patients 2013–2014^a^

Variables	Comparison group	ECHO pain group*
Patients seen per year	1,187,945	52,941
Sex		
Female	39.9%	54.6%
Male	60.1%	45.4%
Age		
18–24	27.1%	19.7%
25–34	33.7%	30.6%
35–44	19.0%	21.4%
45–64	20.2%	28.3%
Beneficiary category		
Dependents of active duty	23.2%	37.2%
Retired	10.1%	14.3%
All others	11.6%	15.5%
Active duty and guard/reserve	55.1%	33.0%
Provider participation level in ECHO		
Low (1–3 TeleECHO clinics)	N/A	47.7%
Medium (4–19 TeleECHO clinics)	N/A	32.1%
High (> 20 TeleECHO clinics)	N/A	20.2%

^a^The number of patients for non-ECHO Pain and ECHO Pain at baseline (pooled 2013–2014) represent annualized totals on the flow diagram (Fig. 2)

Comparison = adult patients enrolled with PCC who did not participate in ECHO Pain; ECHO Pain = adult patients enrolled with PCC who participated in ECHO Pain

*Variables sex, age, and beneficiary category differ significantly between ECHO Pain and the Comparison groups (all*P* < 0.001) possibly due to the large sample size indicated by patients seen

Baseline demographics of adult beneficiaries included approximately 24,000 males (45.4%) and 29,000 females (54.6%) whose PCCs participated in ECHO Pain. The comparison group included 721,000 males and 479,000 female whose PCCs did not participate in ECHO Pain. Age distribution of adult beneficiaries for ECHO Pain was 18–24 years: 19.7%; 25–34 years: 30.6%; 35–44 years: 21.4%; and 45–64 years: 28.3%, and the corresponding age distribution for comparison group was 27.1%, 33.7%, 19.0%, and 20.2%. Fifty-two percent of PCPs participated in four or more TeleECHO clinics (see Table 1).

### Opioid Prescriptions per Patient

**Fig. 3:**
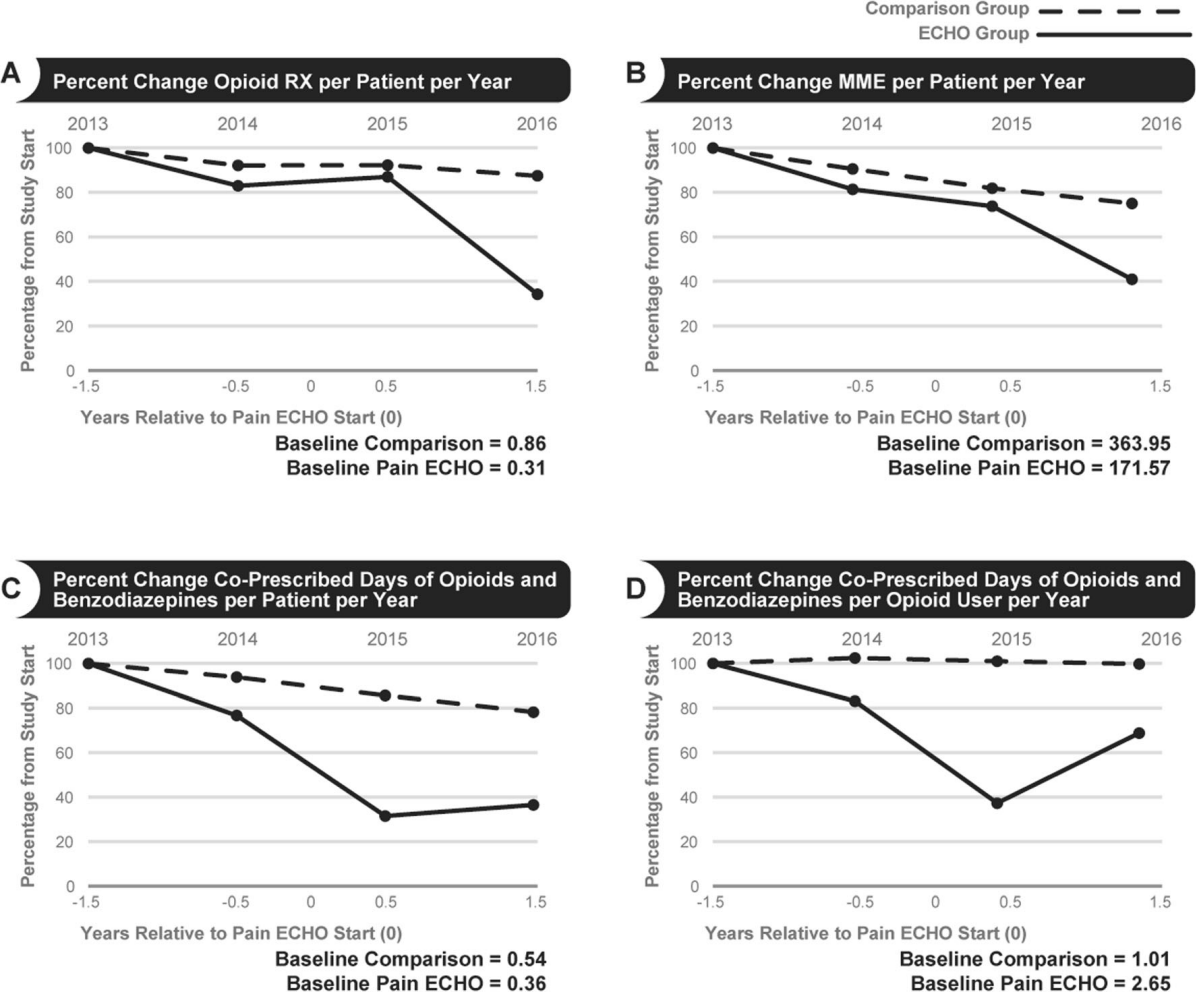
Percent change for selected outcome measures.

While prescribed opioid rates declined in both comparison clinics and ECHO Pain (both *p* < 0.001), the relative decline was greater in clinics participating in ECHO Pain (Fig. 3a). The average annual percent declines were 9.2% (*p* < 0.001) from a baseline of 0.86 RX/patient/year in comparison clinics and 23.0% (p < 0.001) from a baseline of 0.31 RX/patient/year in ECHO Pain. The slopes differed between ECHO Pain and comparison groups (*p* = 0.004, Table 2).

**Table 2:** The Effects of ECHO Pain Clinician Participation on Prescribing of Opioids and Co-prescribing of Opioids and Benzodiazepines

Theme/variable	Comparison baseline	ECHO Pain baseline	Comparison annual change percentage, *P* value	ECHO Pain annual change percentage, *P* value	Interaction *P* value
Opioid analgesic prescriptions (Rx)					
Avg. number of opioid RX/patient/year	0.86	0.31	− 9.2, *P* < 0.001	− 23.0, *P* < 0.001	0.004
Avg. number of opioid RX/opioid user/year	1.61	2.27	− 0.6, *P* < 0.001	− 1.8, *P* = 0.36	0.41
Percent opioid users	53.56	13.69	− 8.0, *P* < 0.001	− 20.2, *P* < 0.001	< 0.001
Morphine milligram equivalents (MME)					
Avg. MME/patient/year	363.95	171.57	− 7.3, *P* < 0.001	− 28.0, *P* = 0.002	0.02
Avg. MME/opioid user/year	679.53	1253.14	+ 1.1, *P* = 0.30	− 6.2, *P* = 0.33	0.32
Percent opioid users	53.56	13.69	− 8.0, *P* < 0.001	− 20.2, *P* < 0.001	< 0.001
Co-prescribing opioids and benzodiazepines					
Days of co-Rx/patient/year	0.54	0.36	− 9.6, *P* < 0.001	− 68.9, *P* < 0.001	< 0.001
Percent patients who are co-Rx users	3.26	1.22	− 8.5, *P* < 0.001	− 68.9, *P* < 0.001	< 0.001
Days of co-RX/opioid user/year	1.01	2.65	− 1.3, *P* = 0.41	− 53.3, *P* = 0.002	< 0.001
Percent opioid users who are co-Rx users	6.09	8.92	− 0.2, *P* = 0.82	− 26.6, *P* = 0.002	< 0.001

Average annual percent change over the study period in comparison and ECHO Pain groups for several outcome measures concerning opioid use (listed in the left column). Average annual change refers to regression slopes per year in the Repeated Measures (RM) ANCOVA analyses; negative slopes indicate decline. Average baseline values (of the outcome variable listed in the corresponding row) are in the second and third columns for comparison and ECHO Pain groups, respectively. The fourth and fifth columns contain the annual % changes (slopes) and *P* values testing whether there was any change over time within a group. The final column contains the interaction *P* values testing whether the two slopes for comparison and ECHO Pain groups differ and indicating whether the ECHO Pain intervention was effective

### MME Dosages per Patient

While prescribed MME dosages declined in both comparison clinics and ECHO Pain, the relative decline was greater in clinics participating in ECHO Pain (Fig. 3b). The average annual percent declines were 7.3% (*p* < 0.001) from a baseline of 364 MME/patient/year in comparison clinics and 28.0% (*p* = 0.002) from a baseline of 172 MME/patient/year in ECHO Pain. The slopes differed between ECHO Pain and comparison groups (*p* = 0.02, Table 2).

### Co-prescribed Opioids and Benzodiazepines per Patient

While days of co-prescribed opioids and benzodiazepines declined in both comparison clinics and ECHO Pain, the relative change was greater in clinics participating in ECHO Pain (Fig. 3c). The average annual percent declines were 9.6% (*p* < 0.001) from a baseline of 0.54 co-Rx/patients/year in comparison clinics and 68.9% (*p* < 0.001) from a baseline of 0.36 co-Rx/patient/year in ECHO Pain. The slopes differed between ECHO Pain and comparison groups (*p* < 0.001).

### Co-prescribed Opioids and Benzodiazepines per Opioid User

While days of co-prescribed opioids and benzodiazepines per opioid user declined in both comparison clinics and ECHO Pain, the relative change was greater in clinics participating in ECHO Pain (Fig. 3d). The average annual percent declines were 1.3% (*p* = 0.41) from a baseline of 1.01 co-Rx/opioid user/year in comparison clinics and 53.3% (*p* = 0.002) from a baseline of 2.65 co-Rx/opioid user/year in ECHO Pain. The slopes differed between ECHO Pain and comparison groups (*p* < 0.001, Table 2).

### Percent Opioid Users

While the percent of opioid users in their patient panels declined in both comparison clinics and ECHO Pain, the relative decline was greater in clinics participating in ECHO Pain. The percent of opioid users in comparison clinics declined annually at 8.0% (*p* < 0.001) from an average annual baseline of 53.6%. The percent of opioid users in ECHO Pain declined annually at 20.2% (*p* < 0.001) from a baseline of 13.7%. The slopes differed between ECHO Pain and comparison groups (*p* < 0.001, Table 2).

### Alternative Analysis

Since baseline values of outcome variables were higher for the comparison group, a potential selection bias, an alternative analysis was conducted to adjust for such a bias. Alternative analyses for percent opioid users, average number of opioid prescriptions per patient/year, and average MME/patients/year used propensity scoring transformations. Resulting weights were applied to the comparison group data, attempting to reduce the selection bias present at baseline. Results were adjusted for age, sex, and beneficiary status as covariables (see SOM). Baseline bias was reduced, and since the alternative analysis results differ little from the original results, they validate the original analysis (see Table 3 for primary outcome variables).

**Table 3:** The Effects of ECHO Pain Participation on Prescribing: Results Adjusted for Selection Bias and for Sex, Age, Beneficiary Category Covariables (Alternative Analysis)

Theme/variable	Comparison baseline	ECHO Pain baseline	Comparison annual change percentage, *P* value	ECHO Pain annual change percentage, *P* value	Interaction *P* value
Opioid analgesic prescriptions (Rx)					
Avg. number of opioid RX/patient/year	0.56	0.31	− 6.4, P < 0.001	− 22.5, *P* < 0.001	< 0.001
Percent opioid users	29.0	13.5	− 8.0, P < 0.001	− 20.1, *P* < 0.001	< 0.001
Morphine milligram equivalents (MME)					
Avg. MME/patient /year	362	176	− 10.6, P < 0.001	− 27.5, *P* = 0.002	< 0.001
Percent opioid users	29.0	13.5	− 8.0, P < 0.001	− 20.1, *P* < 0.001	< 0.001

Alternative analysis using a propensity scoring transformation with the ECHO distribution as target, i.e., ECHO Pain is unchanged but the comparison group is weighted attempting to reduce the selection bias (see SOM). The addition of sex, age, beneficiary category to model adjusts for lack of balance in Table 1. Importantly, annual change percentages are little affected validating the original analysis. These alternative analyses also do reduce baseline bias

## DISCUSSION

Recent Centers for Disease Control and Prevention (CDC) and Veteran’s Affairs (VA)/DoD guidelines recommend safe opioid prescribing practices, especially focused on moderate- and high-dose opioids (> 50 MME) and opioids in combination with benzodiazepines.^3, 32^ Findings from this study provide evidence that ECHO Pain may be used as a successful tool for effectively teaching PCCs to apply safe opioid prescribing practices.

Since the launch of ECHO Pain in 2012, many other interventions nationwide have been implemented to address the opioid epidemic. These include the National Pain Strategy, CDC Pain Management Guidelines, and the VA/DoD Opioid Guidelines, as well as the military sole provider program.^1, 3, 32–34^ These interventions may explain why*both* ECHO Pain PCCs and the comparison group demonstrated declines in opioids and co-prescription.

Clinicians who volunteered to participate in ECHO Pain had lower rates of opioid prescribing and opioid/benzodiazepine co-prescribing at baseline, with higher average baseline MME. We postulate that this difference reflects clinicians who were early adopters of best practices for pain management and who may have treated more challenging chronic pain patients. Both ECHO Pain and comparison groups had declines in opioid prescribing. ECHO Pain had steeper declines than the comparison group even though it started at a lower baseline. ECHO Pain PCCs may have had more initial interest in learning about complex chronic pain patients and developed self-efficacy in managing these patients by participating in ECHO Pain.^26^ Repeat exposure to ECHO Pain deepens knowledge, skills, and confidence, and may explain the robust reduction in opioid prescribing demonstrated in this study.^35^

The significant findings of this study provide evidence that interventions such as ECHO Pain may contribute to reductions in opioid prescribing and co-prescribing of benzodiazepines. ECHO Pain incorporates case-based learning and didactics, promotes increased PCC self-efficacy, knowledge development, and increases social connectedness among participants.^36, 37^

Fifty-two percent of the PCCs exposed to ECHO Pain in this study participated in ≥ four training sessions. This is similar to the ECHO Pain dose-response seen in a recent large Veterans administration SCAN-ECHO Pain analysis showing improved quality of pain care.^38^ Future data analyses may identify the necessary minimum exposure to ECHO Pain which allows PCCs to acquire the knowledge and skills necessary for successful prescribing changes seen in this study. Learning collaborative strategies posit that iterative educational processes are necessary to reduce variation in care and promote practice change.^39–41^

Regardless of the educational platform (multi-point videoconferencing or live trainings), many key stakeholders agree that clinician education in pain and opioid prescribing should focus on providing more non-pharmacologic and non-opioid pain management tools for PCCs while assuring that opioids are prescribed safely when indicated.^3, 35, 42^ CME in pain management and safe opioid prescribing is associated with reductions in opioid and benzodiazepine dispensing as well as reductions in overdose mortality.^43, 44^ There is growing clinician and policymaker support for mandated pain and addiction education.^42, 45–47^ Clinicians practicing in the military (DoD), the Veterans Affairs, and the Indian Health Service (HIS) as well as 23 states and the District of Columbia now have mandatory CME requirements for pain and safe opioid prescribing.^48^

## LIMITATIONS

This study has limitations related to data analysis and selection bias. Most importantly, selection bias may explain the results because of several naturally occurring factors. Because this study could not randomize the assignment of clinicians and patients into matched groups, the baseline demographics for the comparison group are skewed towards male and active duty patients. To the extent that these show up in baseline outcome values, our alternative analysis with propensity scoring adjusts for this. In addition, PCCs volunteered to attend ECHO Pain and, despite the fact that their patient panel appeared to include highly complex chronic pain patients on high doses of opioid analgesics, this clinician cohort may have skewed the results.

The database is a de-identified, aggregated file from the MDR and clinician ECHO Pain participation data provided by the Army and Navy. Due to the aggregated nature of the data, it was not possible to analyze the data down to the individual clinician or patient level. Data was provided on individual clinics with MTFs, but not on individual providers or patients.

Due to the aggregated nature of the data, the authors could not specify the reasons opioids or benzodiazepines were used in each patient. Additionally, it was not possible to (1) quantify how each patient’s opioid MME dose may have changed or (2) to address specific patient/level causes for the reductions in opioid prescriptions, co-prescribing, and MME.

## CONCLUSION

The findings suggest that the ECHO model may be effective both in reducing opioid-related prescription rates and in training PCCs. ECHO Pain reduces variation in care, promoting case-based learning with short, pertinent, evidenced-based didactics. This study suggests that clinician education in both the federal and civilian sectors may have a substantial impact on the opioid epidemic. Since ECHO Pain in this study represented only 5% of the beneficiary population, ECHO Pain could be expanded to benefit a greater number of patients. Additionally, future prospective clinical trials with chronic pain and/or other common and complex conditions may provide information regarding the benefits of the ECHO model at the patient level.

## Electronic supplementary material

ESM 1 (DOCX 172 kb)
