# Supplementary material for: Army and Navy ECHO Pain Telementoring Improves Clinician Opioid Prescribing for Military Patients: an Observational Cohort Study
Source: J Gen Intern Med. 2018 Oct 31;34(3):387–95. doi: 10.1007/s11606-018-4710-5 (PMC6420488; doi:10.1007/s11606-018-4710-5)
Supplement: Supplementary file 1 — (DOCX 172 kb) [file 11606_2018_4710_MOESM1_ESM.docx]

**SUPPLEMENTAL ONLINE-ONLY MATERIAL (SOM)**

**Title: Army and Navy ECHO Pain Telementoring Improves Clinician Opioid Prescribing for Military Patients**

**Authors:**

Joanna G. Katzman, MD, MSPH

Clifford R. Qualls, PhD

CAPT William A. Satterfield, PhD

Martin Kistin, MD

Keith Hofmann, BS

Nina Greenberg, MA, MPH

Robin Swift, MPH

George C. Comerci, MD, FACP

Rebecca Fowler, MPH

Sanjeev Arora, MD, FACP

**Preamble / Introduction**

Supplemental eMethods address the less well known/understood concepts and interpretations in this analytical approach.

**Contents of the Supplemental Online-Only Material (SOM)**

**eMethods**

**Aggregation**

The theory of aggregation

**Definition of the Time Line**

eFigure 1

Why take the logarithm of rates and proportions for model computations?

Fix the distribution by using log values

Use natural log values to estimate percent change in rates and proportions

**Propensity Scoring versus Direct Standardization**

Direct standardization

Propensity scoring

Identifying propensity scoring with direct standardization

Use of baseline outcome values in propensity scoring or direct standardization

Propensity scoring/direct standardization with aggregate data

Does propensity scoring eliminate the need to adjust for covariables?

A limitation of propensity scoring or direct standardization in time series

**Supplemental Analysis**

**Comparison of Means Before and After Intervention in ECHO Pain**

eTable 1

**eMethods**

**Aggregation**

The database for this study is aggregated, which we use here to illustrate the concept of aggregation. This military (Army, Navy) medical clinical database is derived from detailed patient pharmacy records of opioid and benzodiazepine (and other drug) prescription data including date the script was written, and from patient demographic information, and linked to information for clinics and primary care clinician (PCC) teams in clinics. We consider each row (record, observation) of this aggregated database to be a strata. The stratification factors are time (4 years) and PCC team (many) with additional stratification for sex (2), age category (4), beneficiary category (4), and military service (Army and Navy). Beneficiary refers to the military health care plan. The PCC teams are selected into two groups; those participating in the Army and Navy Chronic Pain and Opioid Management TeleECHO Clinic intervention (ECHO Pain group, 99 teams) and those not participating (comparison group, 1183 teams). We call each PCC team a clinic even though the team may not represent the whole clinic. For each row (strata), the data is aggregated into the sum of the number of opioid scripts, and milligram morphine equivalent (MME) doses (and the same is done for other drug scripts) and the number of patients in panels summed over the PCCs in that clinic that year. Addition stratification factors for ECHO Pain teams are a binary indicator of before/after the date of ECHO Pain intervention and a trinary factor of the level of PCC team participation in the ECHO Pain intervention.

Our aggregated database could have more than 328,448 (=4x1282x2x4x4x2) rows (strata) except that several are missing. Our database presents the possibility of aggregating to higher levels such as to the clinic level or even the group level. There is also the possibility of aggregating to the “margin” such as retaining the age classification (the margin) while summing over all of the other covariable classifications. A central idea to computing rates and proportions in each type of aggregation is to track the denominators as well as the numerators of the rates.

Purposes of aggregation might be to reduce the size of the database or to de-identify sensitive data. Another important purpose of aggregation might be to “smooth” the data eliminating some noise and making the “signal” easier to determine. Averaging has this property of smoothing and summing is analogous; noted that rates and proportions are averages. Finally, the purpose of the database is for the computation of valid, often average, quantities suitable for reporting. All of these apply to our study database.

**The theory of aggregation.** Aggregation is justified by the Law of Total Expectations from Probability Theory:

1. $E\left( Y \right)=E_{X}E\left( Y | X \right) ,$

where Y, X are random variables, E(Y) is the expectation of Y and E(Y|X) is the conditional expectation of Y given values of X.

The expectation E_x_ is the expectation with respect to the distribution of X. If the distribution of X were discrete taking values x_i_ with$\sum_{i=1}^{k} P\left( X=x_{i} \right)=1$, then equation 1) becomes

1$'$) $EY= \sum_{i=1}^{k} E(Y|X=x_{i}) P(X=x_{i})$, which explain the word “total”.

Note that k can be finite or infinite. Further if Y = I_A_ , the indicator random variable of the event A, then 1’) becomes the Law of Total Probabilities.

$$1'') P(A)=\sum_{i=1}^{k} P(A\left| X=x_{i} \right)P(X=x_{i}) .$$

Of interest, our study of rates is covered by equation 1$'$) and of proportions is covered by equation 1$''$). The connection between rates and proportion is a reason for these two concepts to be included in the 1972 classic text by Joseph L. Fleiss entitled “Statistical Methods for Rates and Proportions”.

To illustrate the application of equation 1$'$), consider the computation that aggregates one level to a higher level. Let Y = the rate of opioid scripts per beneficiary, and the binary X representing the 2 clinic teams. Then the conditional expectation (weighted mean) is E(Y|X=i) = r_i_ , the rate specific to the teams i=1,2 and P(X=i) is the relative frequency of the two teams in the database. Then the overall rate r by 1$'$) is r = EY = $\sum_{i=1}^{2} E\left( Y | X=i \right)P\left( X=i \right)= \sum_{i=1}^{2} r_{i} P\left( X=i \right)$.

Note the slight difference in that aggregation tracks the numerators and denominators separately, but equation 1$'$) calculations are about the ratios of these.

The authors used the Law of Total Expectations in this paper to

1) verify that averages, rates and proportions are treated alike in computations in aggregated databases, and

2) to track the computations across multiple levels of aggregation; for example, computations in aggregation of data in strata to the clinic level and then to the group level.

**Definition of the Time Line**

The staggered starts of the ECHO Pain intervention often in mid-fiscal year result in unbalanced before and after fiscal years (**see eFigure 1 below**). So a straight forward analysis by fiscal year is not optimal. For example, 75 of the 99 clinic PCC teams begin in mid-fiscal year so that this fiscal year has data for both before and after the start of the ECHO Pain intervention. In these cases, a study by fiscal year would pool before/after data that may be different and are different according to our research hypothesis. This seems an inadequate method for testing this hypothesis and is a reason for defining a time line that acknowledges this problem. The following schematic diagram illustrates this definition based on midpoints of periods.

eFigure 1


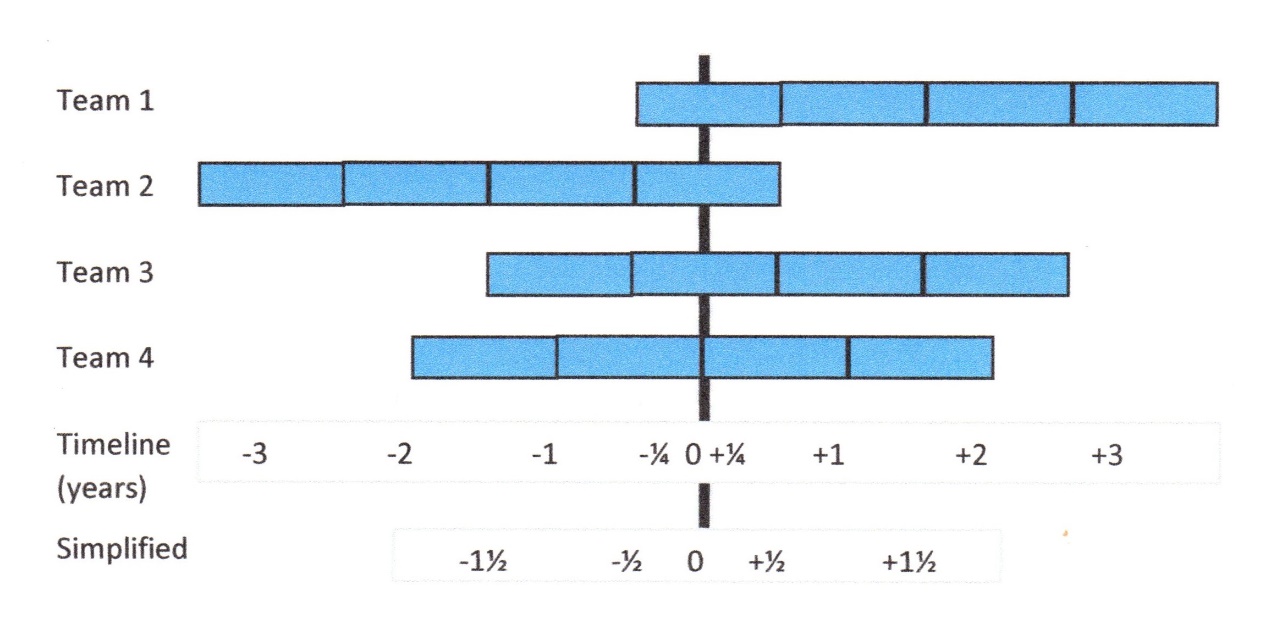


**eFigure 1**. Schematic diagram of time line for the ECHO Pain intervention. The start of ECHO Pain is represented by the heavy black, vertical behind the 4 consecutive fiscal years, which are represented by the 4 blue adjacent rectangles for each team. If the start of ECHO Pain is mid-year as in teams 1-3, then that fiscal year is divided into a before half year and an after half year (half year is an approximation) and midpoints of each half year is represented as – ¼ and +¼ on the time line. All of the comparison (non-ECHO Pain) group teams and for 25% of the ECHO Pain teams are presented by team 4, where the start is at the beginning of the year. The use of midpoints for the fiscal years for these teams give a simple time line (see Simplified in eFigure 1); the full time line is needed for remaining 75% of the ECHO Pain teams and are represented by Teams 1-3 in **eFigure 1**. We delete years -3 and +3 for reasons 1) years ± 3 are higher leverage points (extreme values on the x-axis) in regression as a function of time, 2) years ± 3 are the farest from the ECHO Pain start and might be the least related to the ECHO Pain intervention, and 3) in addition, years ± 3 represented a small percentage of the beneficiaries. Midpoints are used to obtain regression slopes in **Table 2**. The simplified time line is used for graphical purposes (**Figure 3**).

**Why take the logarithm of rates and proportions for model computations?** There were two reasons to take the logarithm of rates and proportions; 1) to deal with outliers and to make the distributions more symmetric, and 2) regression of the natural logarithm of rates and proportions provides direct computation of the percent change in rates and proportions over time before and after the start of ECHO Pain. We use natural logs throughout because of reason 2. In general, there are 3 reasons statisticians give for using log-values: a) to make the distribution symmetric (more normally distributed) and down regulate outliers, b) to linearize the relation between Y and X as log(Y) versus X, and c) to equalize variances in the common situations where the variance (or standard deviation) increases with mean of Y; many statistic procedures (e.g., ANalysis Of VAriance, ANOVA) work better with equal variances. We emphasize reason a).

**Fix the distribution by using log values.** First there were outliers and non-normal distributions of rates and proportions. A usual solution to try is to use logarithm-values. Side-by-side box plots of our outcome data show that logarithm transformation will help. A remaining problem is that the logarithm of zero rates and proportions in several strata become missing despite the fact that the zeros represent important information. The usual solution is to take log (Y+1) so that log (0+1) =0. In our data, there is a noticeable gap between 0 and the minimum non-zero values which is often made larger by the log transformation (for rates < 1). To obtain transformed distributions nearer to normal distributions we take log (Y+c) where c = 90% of the minimum positive value for each outcome in each group (ECHO Pain and comparison).

**Use natural log values to estimate percent change in rates and proportions.** This idea is based on the calculus of derivatives where the differential Δ ln(Y) ≈ ΔY / Y . So the regression of ln(Y) versus t in years estimates the regression coefficient, which is a slope of the form b = Δ ln(Y) / Δt. Here, the Δt is 1 year so the slope b = Δ ln(Y) ≈ ΔY / Y, which is the relative change in Y per year. And 100* ΔY / Y is the annual percent change in Y. Note that ΔY can be positive or negative for increasing or declining Y, respectively. If ΔY = 0, then there is no change and Y is constant. (This annual percent change is analogous to an annual interest rate.) Thus, if this annual percent change in Y were applied over the 3 intervening years of our 4 year study, then the total change would be obtained by compounding the instantaneous annual percent change rate over the 3 years. This total change can computed approximately using the multiplicative factor (1+ ΔY / Y)^3^ . For example, if Y is declining at a rate of 15% per year, then over the 3 intervening years the rate will decline approximately 52% since (1 +0.15)* (1 +0.15)* (1 +0.15) = 1.52. It should be pointed out that %changes are comparable even when beginning differences are present and even when absolute changes differ; this is the idea of reporting annual interest rates in financial comparisons.

**Propensity Scoring versus Direct Standardization**

**Direct standardization.** We begin by explaining direct standardization; it is a simpler concept than propensity scoring which we explain second. A typical example involving direct standardization consists of two (2) populations, whose age distributions are f_1_ and f_2_, and a target age distribution g, say the 1990 U.S. Census standard age distribution. Here age is considered a predictor variable of major effect (analogous to a gene of major effect on some trait in the field of genetics). The basic computation mapping f_1_ to g is simply explained by the arithmetic formula g = f_1_ * (g/f_1_), and mapping f_2_ to g by g = f_2_ * (g/f_2_). So one forms the weights w_1_(x) = g(x)/f_1_(x) and w_2_(x) = g(x)/f_2_(x), where x=age, and use weighted analyses in the comparisons of outcome variables Y between population (or between samples from these populations). Of course these computations will make the mean ages in the two populations equal, so the important comparisons are no longer X=age but the outcome variables Y in the study. None-the-less we check the effect of standardization on X=age and thereby give an example of a weighted analysis. Thus, let us compute the adjusted means of X= ages.

$E_{1}\left( X | weights w_{1} \right)=\sum_{i=1}^{n} w_{1}\left( x_{i} \right)x_{i} f_{1}(x_{i} )$ = $\sum_{i=1}^{n} x_{i} g\left( x_{i} \right)=mean of g , and$

$E_{2}\left( X | weights w_{2} \right)=\sum_{i=1}^{n} w_{2}\left( x_{i} \right)x_{i} f_{2}(x_{i} )$ = $\sum_{i=1}^{n} x_{i} g\left( x_{i} \right)=mean of g .$

So the standardization of f_1_ and f_2_ to target g entails that the adjusted means of f_1_ and f_2_ are both equal to the mean of g, as expected. Thus, what is important is the adjusted analyses of the other variables, Y, in the data.

In this paper, we standardize the comparison group baseline distribution f to the ECHO Pain baseline distribution g as the target. To map f to g use weights w = g/f, so that g = f *w.

**Propensity scoring.** Propensity scores are computed by a full multivariable logistic regression model of the selection of the ECHO Pain or comparison groups with the goal of adjusting for selection bias. The logistic regression model provides a predicted value for each observation, which is the propensity score, the probability that ECHO Pain was selected given the set of predictor values for that observation. The concept for before-after intervention time series is that the baseline outcome variable needs to be adjusted so that the baseline is equivalent for the two groups. The hope is that this adjustment will eliminate or reduce the selection bias and that the remainder of the time series, particularly after intervention, will not be biased due to selection. One way this adjustment is made is by using weighted analyses. To be explicit, let propensity scores be π_i_ be the probability of selecting ECHO Pain, where index i represents the i^th^ observation; then 1- π_i_ is the probability of selecting the comparison group. The propensity scores are a function of the baseline predictor variables and are used to define weights for subsequent analyses of the longitudinal outcome as follows

W(x) = 1/PS or ϑ_1_/ π_i_ for observation i in the ECHO Pain group and

W(x) = 1/(1-PS) or (1- ϑ_2_)/ ( 1- π_i_ ) for i in the comparison group

These are called stabilized “inverse probability of treatment weights” (S- IPTW). It can be shown that putting ϑ_1_ and (1- ϑ_2_) in the numerators, instead of 1, has better stability properties. Here ϑ_1_ =mean of π_i_ over i in the ECHO Pain group and ϑ_2_ = mean of π_i_ over i in comparison group, or 1- ϑ_2_ = mean of 1-π_i_ over the comparison group.

A comparison of these formulas for W(x) to those in direct standardization suggests that the propensity score weights are a result of direct standardization of the propensity scores to a common distribution.

$$W\left( x_{i} \right)=\frac{\vartheta_{1}}{\pi_{i}} =\frac{1/n}{{\pi_{i}}/{\sum\pi_{i}}} for observation i in ECHO Pain$$

and

$$W\left( x_{i} \right)=\frac{{1-\vartheta}_{2}}{1-\pi_{i}} =\frac{1/n}{\left( {1-\pi}_{i} \right)/{\sum\left( 1-\pi_{i} \right)}} for i in the comparison group,$$

Writing the propensity scores are normalized to be a probability distributions and the defined weights that map these distributions into the common uniform distribution via direct standardization, how-be-it, in the propensity score space (logit space). Of course, the weights W(x_i_) are applied to the original data (the x-space).

**Identifying propensity scoring with direct standardization.** A justification for relating propensity scoring with direct standardization is outlined in the previous paragraph. We use direct standardization because it allowed a targeted common distribution that we specify; it is not clear what the targeted distribution in the data space is when one uses propensity score weights. Further research is required

**Use of baseline outcome values in propensity scoring or direct standardization.** One way that outcomes might be equalized at baseline is to include the baseline values of the outcome variable(s) in the logistic regression model or standardization model. Is this acceptable? Many analysts include the baseline value of a longitudinal outcome variable as a predictor in the Repeated Measures (RM) ANOVA of the outcome; they/we argue that this adjusts for unknown differences in the treatment groups (unknown because the difference may not be represented by the x-variables known). The same argument will allow the baseline outcome values to be used in the logistic regression defining propensity scores or in direct standardization.

**Propensity scoring/direct standardization with aggregate data.** Propensity scoring/direct standardization with aggregate data involves additional issues. In our case of aggregated military pharmacy data concerned with opioid use, the aggregation is at the PCC team level but with stratification for time (in years), age (in 4 categories), sex, beneficiary category (in 4 categories), and service (Army and Navy). The stratification also provides the number of beneficiaries in PCC patient panels summarized in each of the strata of each PCC team. The statistical analysis is at the strata level, but imputed to the patient level by using the number of beneficiaries as weights. The main comparison is between PCC teams who participated in the ECHO Pain intervention (Pain ECHO group) and the PCC teams who did not participate (comparison group). There is a selection bias; the baseline values of outcomes were different. Propensity scoring has been an effective way of making sense of the data obtained from studies that are not clinical trials and do not randomly select the treatment each participant is assigned.

However, most discussions of propensity scoring has been for individual data and not for aggregate data. We discuss the later here. In our study there are several issues. First, an issue in an aggregated database is that one already has weights representing the aggregation. In addition, we could have weights from a direct standardization step or weights from the propensity scoring step. Some procedures in the statistical package SAS version 9.4 allow both frequency and weighting variables, which solves some of the problem. Thus one may be able to use the frequency statement for the aggregation weights (frequencies) and the weight statement for the weights from standardization or propensity scoring; there is an option (VARDEF=WDF) that will normalize these weights. A second issue involving the baseline of longitudinal data (time series) is discussed in paragraph below.

**Does propensity scoring eliminate the need to adjust for covariables?** No, demographic variables sex, age, and beneficiary category are not balanced between ECHO Pain and comparison groups before or after a selection bias adjustment with our propensity-like weights. Thus, these covariables are added to the model in the alternative analyses for Table 2B.

Generally, it should be explained that propensity scoring may not adjust for in-balance in these variables even if they are included in the multivariable logistic regression defining the propensity scores as predicted values. The prediction model is of the form $logit\left( x_{1}{,x}_{2},\ldots,x_{k} \right)=\sum_{1}^{k} \beta_{i} x_{i}$ in the log odds space. There is a reduction of dimensionality from k variables to one $y=\sum_{1}^{k} \beta_{i} x_{i} .$ So, a 1-dimensional adjustment may not balance all of the k variables. Though this might be possible if in-balance in every variable were solely due to selection bias; however, the in-balance in some variables may be due the real differences between populations. If one wishes to adjust for such real differences, then the adjustment should be made directly, perhaps by inclusion of covariables in the model. Thus, there are three modeling elements in our alternative analyses presented in Table 2B. The SAS syntax is

Model log_e_ (rate) = group|time sex age beneficiary_cat

Frequency

Weight

where covariables are listed in the model statement, frequency specifies number of patients in each strata, and weight specifies weights given by propensity scoring or direct standardization.

**A limitation of propensity scoring or direct standardization in time series.** A potential difficulty with the analysis choice to using a baseline outcome arises when propensity scoring tends to emphasize extreme values of the baseline outcome in one of the groups creating a “regression to the mean” in the outcome values after the baseline. For example in the 1,283 PCC teams in the comparison group, suppose 99 lowest baseline outcome values were selected by propensity scoring to be matched with the baseline values in the 99 ECHO Pain teams. If the outcome variable is a times series of highly variable values then we tend to select low values that are extreme compared to the mean of the series. In the next time after baseline, the series will tend to move back to its mean subject to the correlation structure of the series. This is a “regression to the mean”. If the distributions of the baseline outcome values in the two groups robustly overlap then this “regression to the mean” problem is minimized. Also selecting our baseline as two years before the start of the ECHO Pain intervention, instead of just the first year, appears to eliminate regression to the mean problems. Thus, including baseline outcome values in propensity scoring or standardization may be done but with caution.

**Supplemental Analysis**

**Comparison of Means Before and After Intervention in ECHO Pain**

This is another alternative analysis that supports the findings in the main text. The previous analyses emphasized slopes over the four years. This analysis emphasizes means, but pools the data for the years before and for the years after time 0 (the time of the ECHO Pain intervention). Means are population means and include many zeros; e.g., the per-patient means include those who are not opioid users (86.3 to 89.8% of the patients are not opioid users).

**eTable 1.** Comparison of averages before and after ECHO Pain intervention (2^nd^ and 3^rd^ columns) for several outcome measures (listed on the left); p-values by Repeated Measures (RM) ANOVA.

| **Theme/Variable** | **Means Before ECHO Pain** | **Means After ECHO Pain** | **RM ANOVA**  **p-value** |
| --- | --- | --- | --- |
| **Opioid Analgesic Prescriptions**  Avg. number of opioid Rx/patient | 0.31 | 0.24 | 0.006 |
| **Morphine Milligram Equivalents (MME)**  Avg. MME / patient | 172 | 151 | 0.06 |
| **Co-Prescribing Opioids & Benzodiazepines**  Days of Co-Rx/patient | 0.36 | 0.12 | < 0.001 |
| **Percent opioid users**  % | 13.7 | 10.2 | < 0.001 |
| **Co-Prescribing Opioids & Benzodiazepines**  Days of Co-Rx/opioid user | 2.7 | 1.2 | < 0.001 |
